# Supplementary figures and images for: First Phylogeny of Bitterbush Family, Picramniaceae (Picramniales)
Source: Plants (Basel). 2020 Feb 21;9(2):284. doi: 10.3390/plants9020284 (PMC7076446; doi:10.3390/plants9020284)

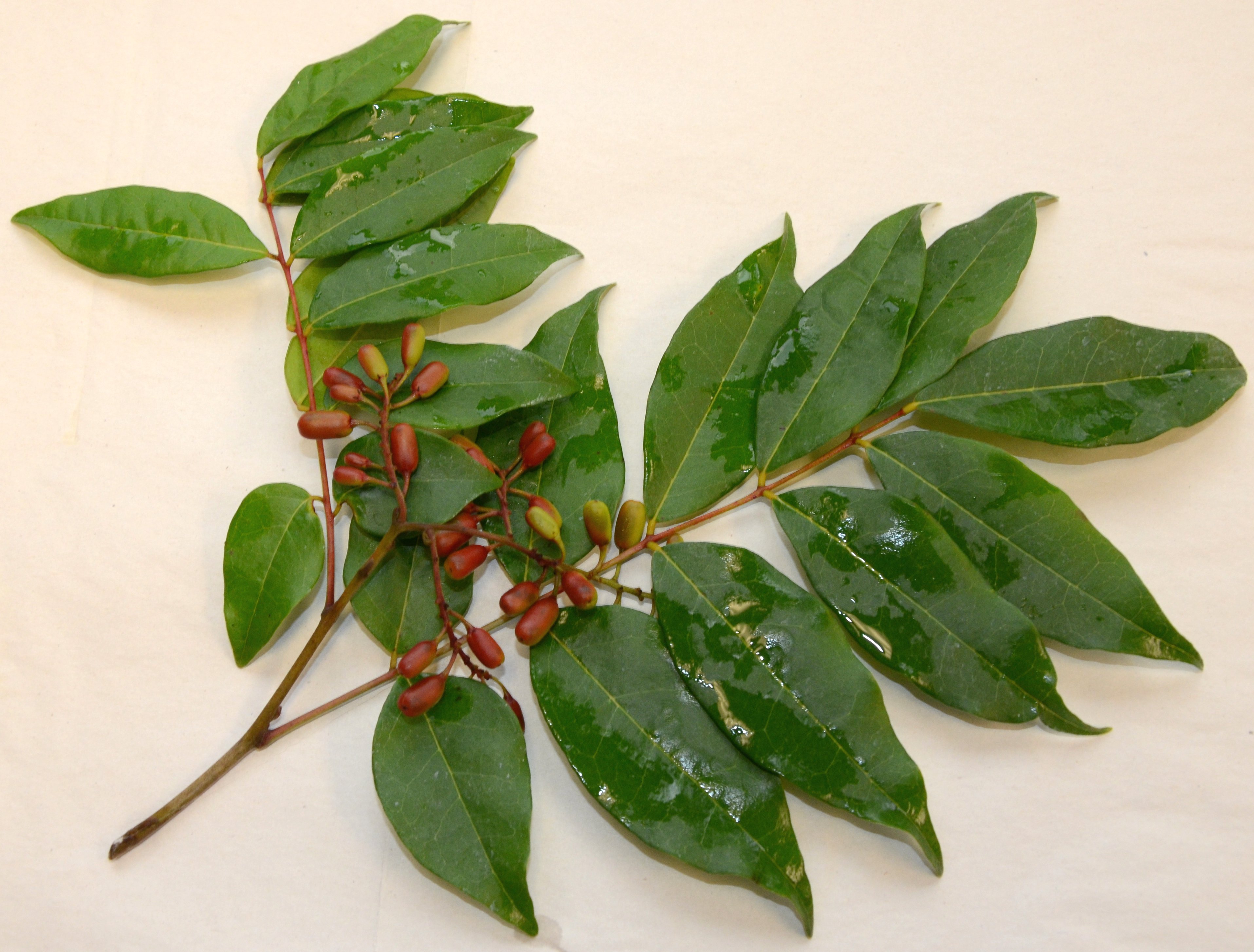

Supplement: Supplementary file 1 [file plants-09-00284-s001.zip › supp_mat/figures/p1_picramnia_pentandra.jpg]

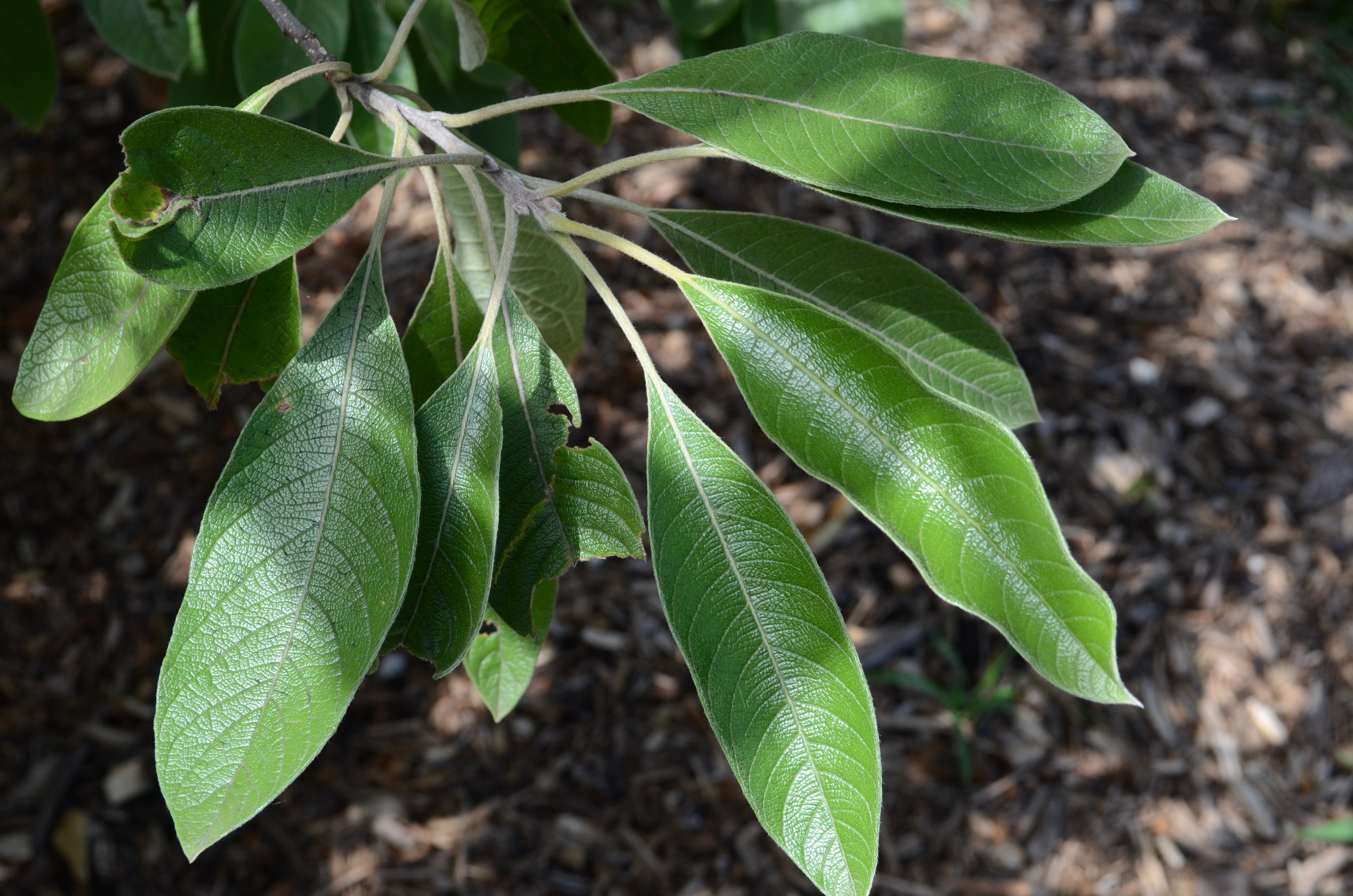

Supplement: Supplementary file 1 [file plants-09-00284-s001.zip › supp_mat/figures/l0_leitneria_floridana_dsc_5921.jpg]

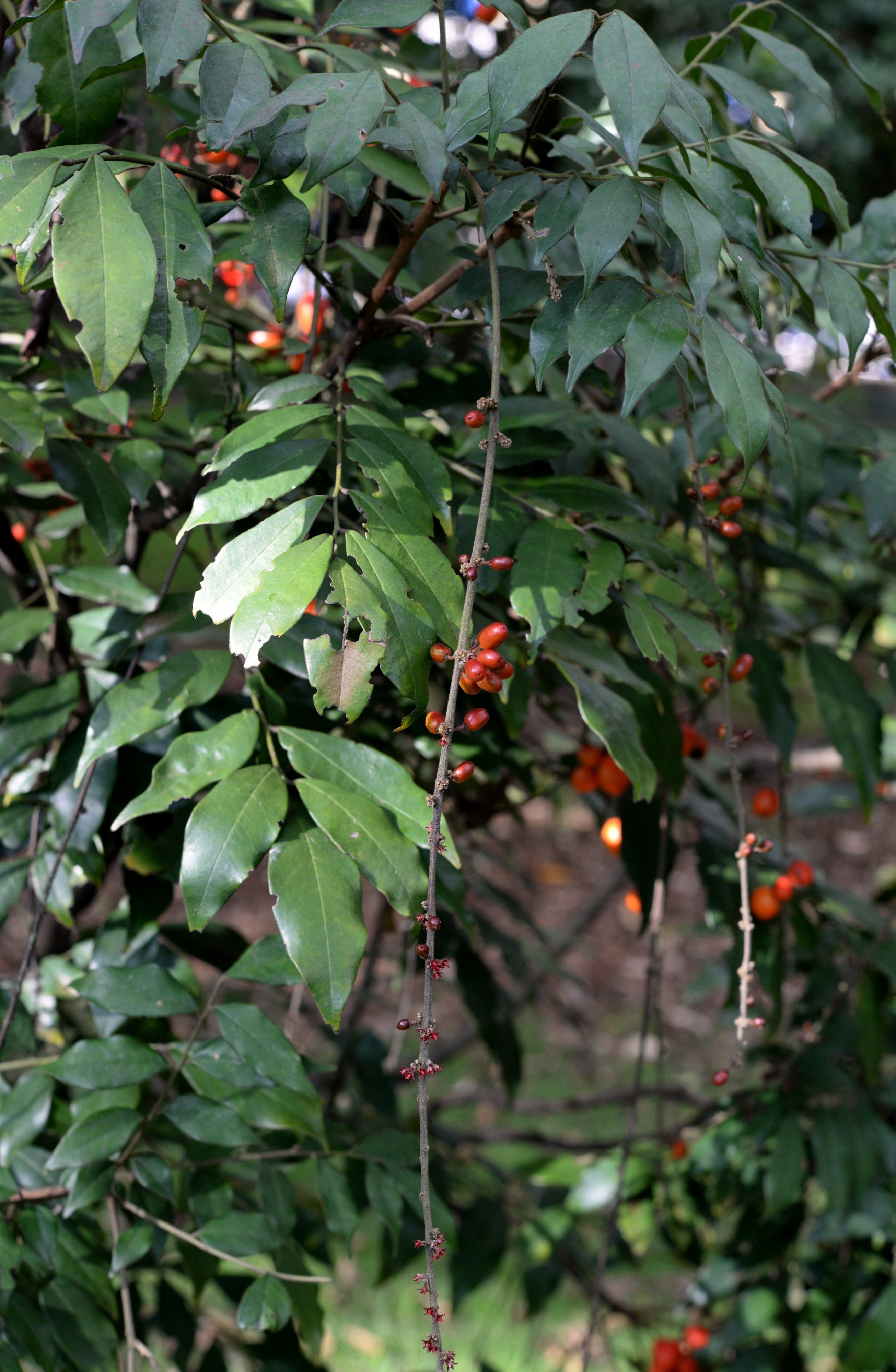

Supplement: Supplementary file 1 [file plants-09-00284-s001.zip › supp_mat/figures/pi_0902_picramnia_sphaerocarpa_abs_8746.jpg]

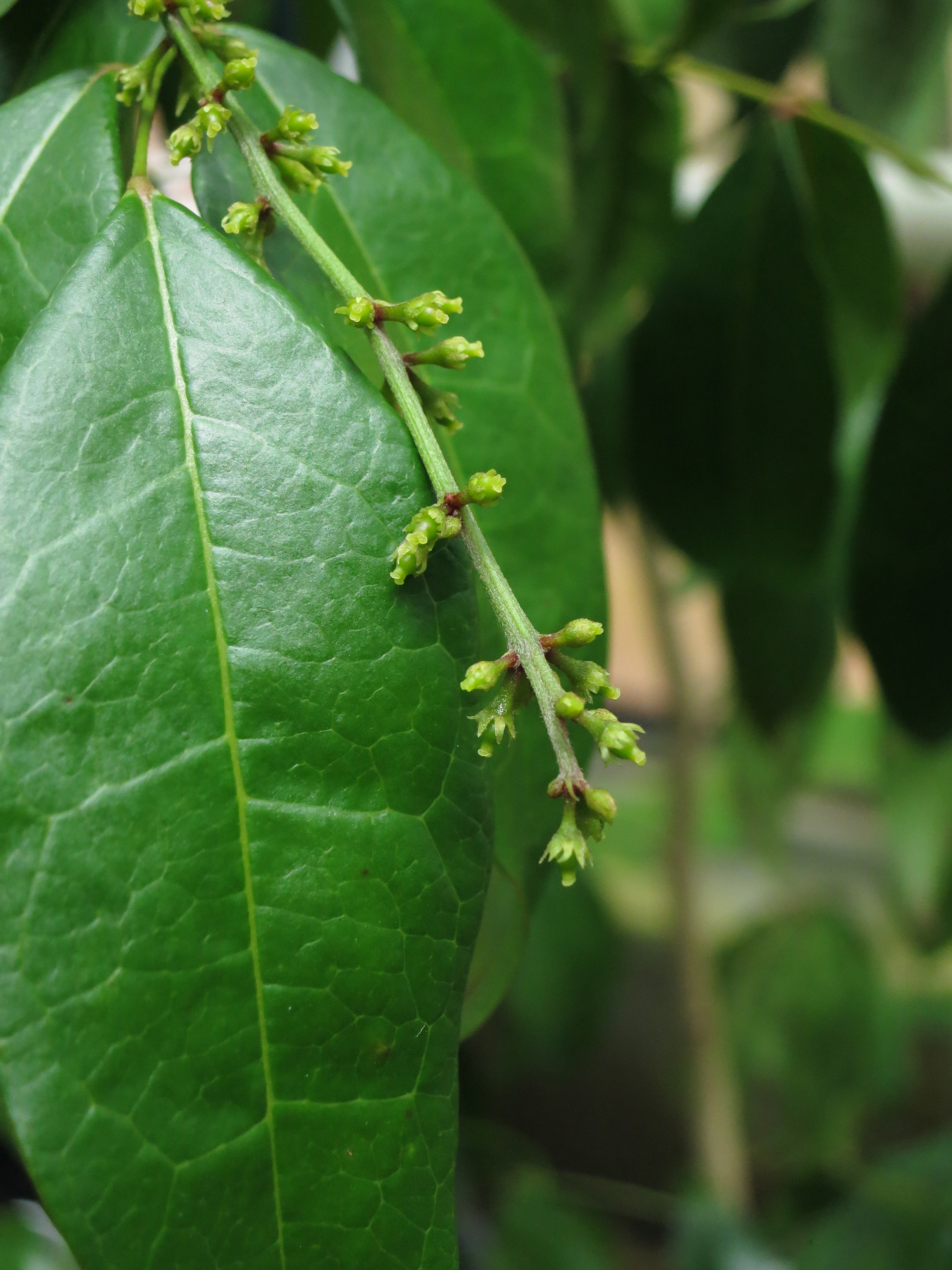

Supplement: Supplementary file 1 [file plants-09-00284-s001.zip › supp_mat/figures/pi_0904_picramnia_pentandra.jpg]

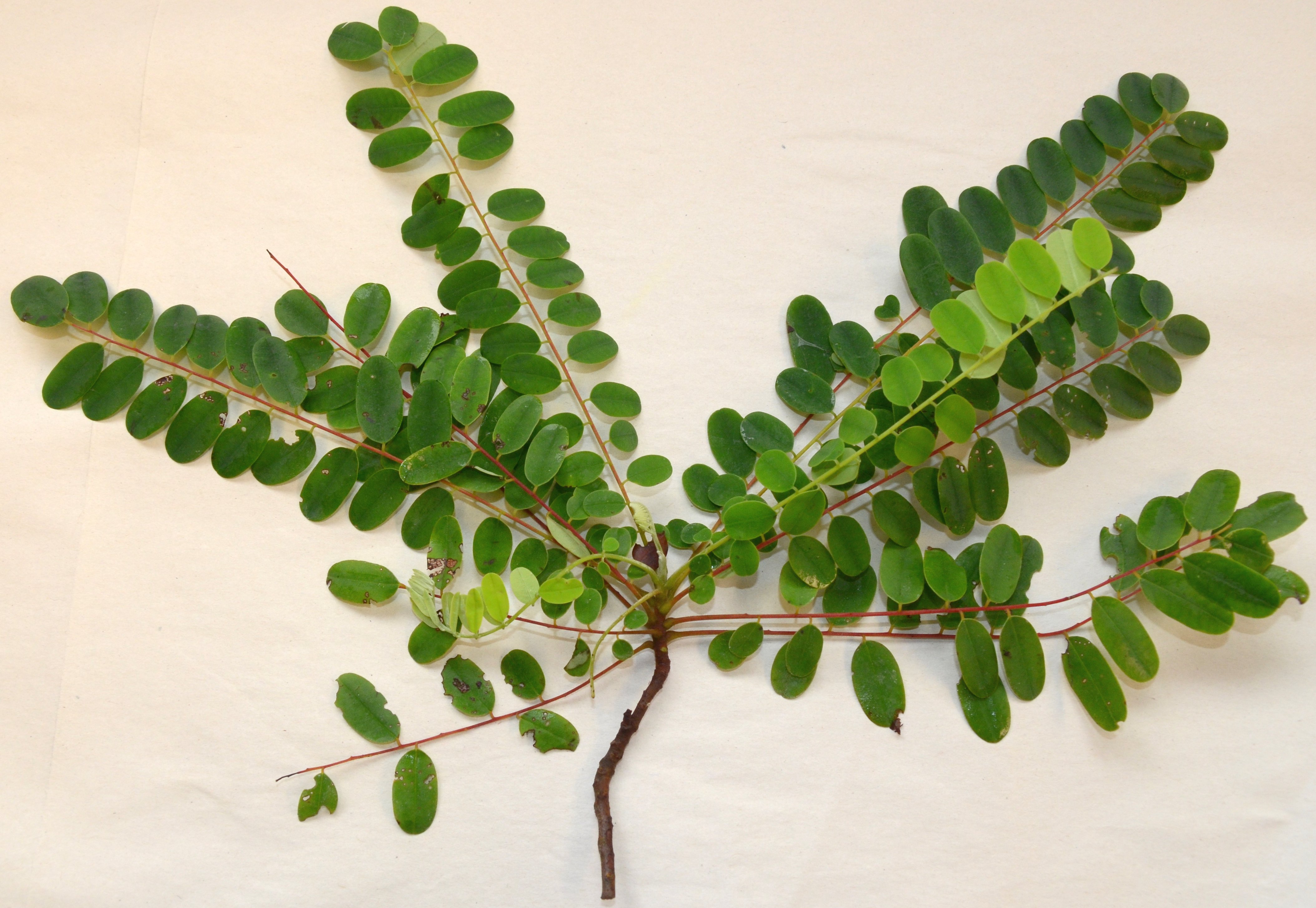

Supplement: Supplementary file 1 [file plants-09-00284-s001.zip › supp_mat/figures/a0_alvaradoa_amorphoides.jpg]

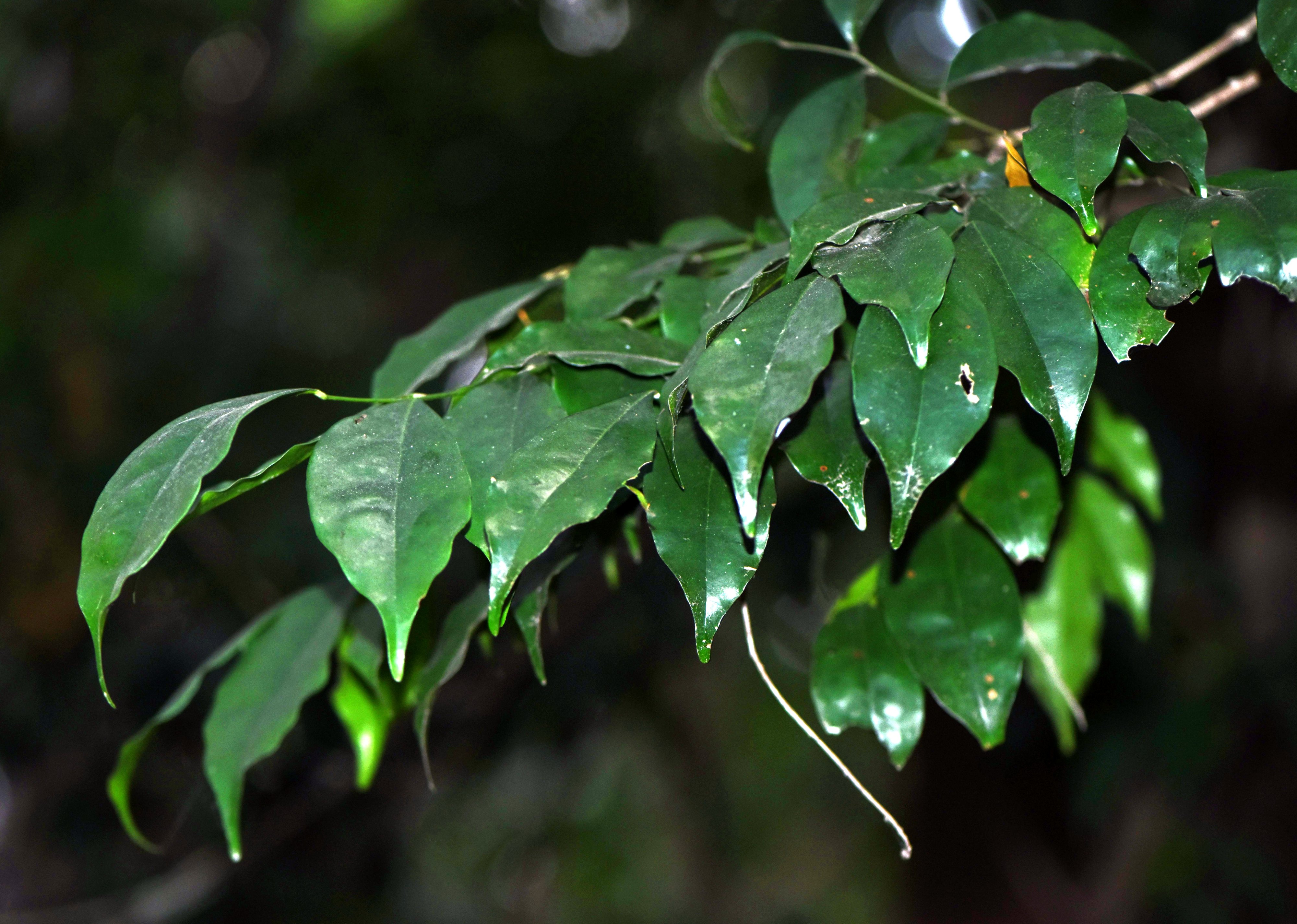

Supplement: Supplementary file 1 [file plants-09-00284-s001.zip › supp_mat/figures/pi_0903_picramnia_glazioviana_ycv_1414.jpg]

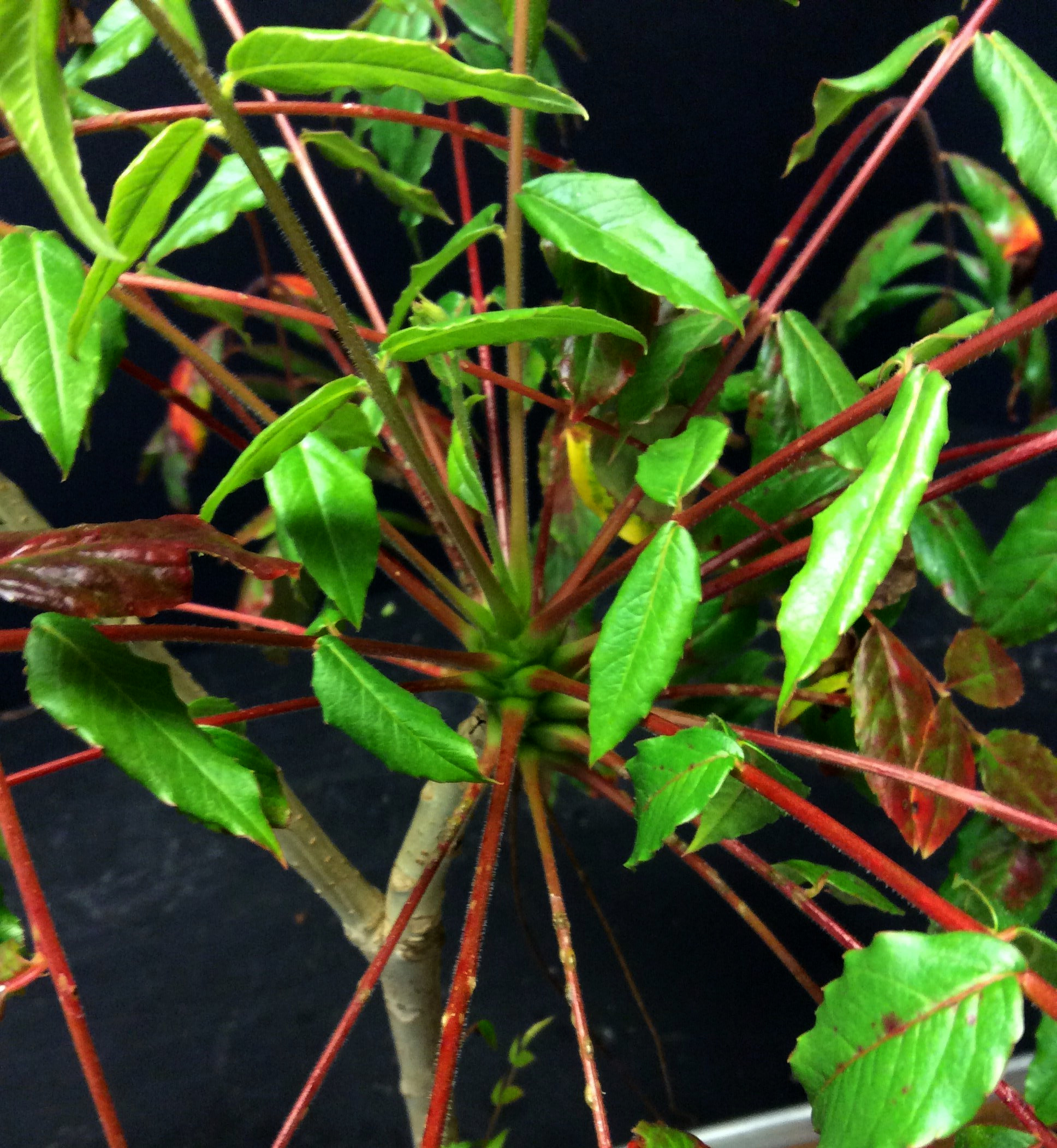

Supplement: Supplementary file 1 [file plants-09-00284-s001.zip › supp_mat/figures/pi_0905_kirkia_acuminata.jpg]
